# Optimal Dosage of Indocyanine Green Fluorescence for Intraoperative Positive Staining in Laparoscopic Anatomical Liver Resection

**DOI:** 10.7759/cureus.46771

**Published:** 2023-10-10

**Authors:** Meidai Kasai, Tsukasa Aihara, Shinichi Ikuta, Takayoshi Nakajima, Naoki Yamanaka

**Affiliations:** 1 Department of Surgery, Meiwa Hospital, Hyogo, JPN; 2 Department of Surgery, Meiwa Hospital, Nishinomiya, JPN

**Keywords:** roc curve analysis, positive staining, indocyanine green, fluorescence imaging technology, laparoscopic anatomical liver resection

## Abstract

Introduction

Fluorescence imaging technology, specifically utilizing indocyanine green (ICG), has emerged as a valuable tool in laparoscopic hepatectomy. In particular, laparoscopic anatomical liver resection (ALR) has benefited from the implementation of both positive and negative staining methods. A case series study reported a success rate of 53% for the positive staining method, citing potential issues regarding the proper ICG dosage needed for accurate fluorescence. Thus, it is crucial to conduct research to investigate the optimal dosage for ICG-positive staining in clinical practice to maximize the benefits of this technique.

Materials and methods

This retrospective study was conducted at a single center, Meiwa Hospital, and received approval from the hospital's ethics committee in accordance with the Helsinki Declaration. We reviewed the records of 264 patients who underwent open and laparoscopic hepatectomies for benign and malignant liver diseases from January 2019 to January 2023. Of these, 18 patients who underwent laparoscopic ALR with the ICG-positive staining method were evaluated. Fluorescence-emitting segmental borders were assessed immediately after puncture (first stage) and during parenchymal dissection (second stage). In the first stage, we evaluated the intensity of fluorescence emission, categorizing it as "strong" or "weak." The absence of visible fluorescence emission was considered a puncture failure. During the second stage of evaluation, from parenchymal resection to completion, we assessed the sustainability of fluorescence emission, defining it as "clear" or "contaminated." Both evaluations were subjectively judged by three surgeons at our center. The ICG quantity per targeted portal vein-bearing liver volume (mg/100 mL) was calculated for each patient, and the optimal dosage was determined using receiver operating characteristic (ROC) curve analysis. To ascertain the minimum value for adequate fluorescence emission intensity, ROC curve analysis was performed to discriminate between binary outcomes of "strong" or "weak" emission. Furthermore, to establish the maximum value for maintaining a clear fluorescence border, ROC curve analysis was conducted to discriminate between "clear" and "contaminated" during the second evaluation.

Results

Among the 18 successful puncture cases, the first-stage evaluation of fluorescence intensity revealed 14 punctures with "strong" intensity and four punctures with "weak" intensity. In the second-stage evaluation, 13 cases demonstrated "clear" borders, while five cases exhibited "contaminated" borders. ROC curve analysis was performed to determine the optimal ICG dose for adequate fluorescence intensity and preservation of clear borders during dissection. The analysis indicated that the appropriate ICG dose for achieving optimal intensity was 0.028 mg/100 mL (area under the curve [AUC]: 0.893), while the dose that prevented contamination of fluorescence in non-target areas until after dissection was 0.083 mg/100 mL (AUC: 0.723).

Conclusions

Laparoscopic anatomical resection using the positive staining method requires an optimal ICG dosage of 0.028-0.083 mg per 100 mL of liver volume. By employing this methodology, more precise and safer laparoscopic anatomical resections can be conducted, thereby enhancing the safety of the surgical procedure for patients.

## Introduction

Fluorescence imaging technology, specifically utilizing indocyanine green (ICG), has emerged as a valuable tool in laparoscopic hepatectomy, demonstrating substantial improvements in operative time, blood loss, hospital stay, and postoperative complications [1]. In particular, laparoscopic anatomical liver resection (ALR) has benefited from the implementation of both positive and negative staining methods, as described by Ishizawa and colleagues, which have played a crucial role in enhancing surgical outcomes [2].

The positive staining method, a variation of Makuuchi's procedure, involves ultrasound-guided portal vein puncture and ICG injection, followed by resection of all visible liver parenchyma upon visualization [2,3]. Conversely, the negative staining method, a variation of Takasaki's techniques, entails a systemic ICG injection after clamping the Glissonian pedicle supplying the tumor-bearing segment, resulting in ischemic, non-fluorescent lesions [2,4]. A case series study by Xu et al. reported a marginally higher success rate of 53% for the positive staining method compared to 51% for the negative staining method. The report cited potential issues such as inappropriate puncture or retrograde ICG flow as factors contributing to staining failure [5]. Concerns were raised regarding the proper ICG dosage needed for accurate fluorescence. Although various studies have addressed the adequacy of dosage in ICG administration, there remains a pressing need to investigate the optimal dosage for ICG-positive staining to maximize the benefits of this technique [5,6,7]. In this retrospective study, we aim to determine the optimal ICG dosage for the positive staining method in laparoscopic anatomical liver resection, based on receiver operating characteristic (ROC) curve analysis from our series of experiences.

## Materials and methods

This retrospective study was conducted at a single center, Meiwa Hospital, and received ethics approval from the Medical Ethics Committee of Meiwa Hospital (No. 2023-21), in accordance with the Helsinki Declaration. Informed consent was obtained from all participating patients. We reviewed the records of 264 patients who underwent open and laparoscopic hepatectomy for benign and malignant liver diseases from January 2019 to January 2023. Cases with incomplete portal vein puncture, which required conversion to the negative staining method, were excluded from the study. All patients underwent preoperative multidetector computed tomography. Three-dimensional (3D) images of the liver, including tumors, vessels, and segmentation, were generated using workstation software (Synapse Vincent; Fujifilm Corporation, Japan). The tumor-bearing portal veins and resected segments were identified, and the predicted resected liver volume was calculated based on the 3D images.

Intraoperative positive staining with ICG

For laparoscopic anatomical liver resection, our institution has standardized the positive staining method as an intraoperative percutaneous ultrasound (US)-guided tumor-bearing portal vein branch puncture and injection of ICG dye under artificial ascites, as previously described [8]. In cases where this approach proves difficult, we opt for laparoscopic intraoperative ultrasound (IOUS) puncture or non-US-guided direct portal vein puncture, particularly for posterior portal branches. During percutaneous portal vein puncture, after liver mobilization, normal saline is injected into the surgical space to displace carbon dioxide, and the patient is positioned in the Trendelenburg position. Subsequently, tumor-bearing portal vein puncture is performed with B-mode ultrasound (Toshiba Medical Systems, Japan). A total of 0.025-0.5 mg of ICG is injected into the target portal vein, and fluorescence-enhanced liver parenchyma is confirmed using the ICG camera system (PINPOINT endoscopic fluorescent imaging system, Stryker, Germany, or IMAGE1 S™ Rubina™, Storz, Germany). Parenchymal dissection with ultrasonic shears is performed along the fluorescence border until the landmark hepatic vein is exposed, and the targeted Glissonian pedicle is divided.

ICG dosage protocol

Our initial cohort of 10 patients was administered an ICG dose of 0.25 mg per liver subsegment, according to the protocol outlined by Ishizawa et al. [2]. However, we observed instances of undesirable segment staining, prompting a revision of the initial dosage to 0.025 mg per liver subsegment in accordance with the methodology proposed by Aoki et al. [7]. In cases where the fluorescence was deemed insufficient, additional punctures were performed to ensure adequate staining.

Evaluation of staining

Fluorescence-emitting segmental borders were evaluated immediately after puncture (first stage) and during parenchymal dissection (second stage), modifying the evaluation strategy reported by Xu et al. [5]. In the first stage, we assessed the intensity of fluorescence emission, categorizing it as "strong" or "weak." Since there was no quantitative method to objectively determine the intensity, three senior surgeons in our center subjectively judged the fluorescence. If they determined it was sufficiently intense for the surgical procedure, it was categorized as "strong." If the fluorescence was perceivable but deemed weak, it was classified as "weak." Cases where fluorescence emission was not visible were considered puncture failures. During the second stage of evaluation, from parenchymal resection to completion, we assessed the sustainability of fluorescence emission, defining it as "clear" or "contaminated." "Clear" was defined as a distinct boundary between the tumor-bearing portal segment and other segments, both on the liver surface and within the liver parenchyma. "Contaminated" indicated that undesired segments gradually became contaminated, or the targeted portal segment became obscured throughout the procedure.

Statistical analysis and determination of optimal ICG dosage

The ICG quantity per targeted portal vein bearing liver volume (mg/100 mL) was calculated for each patient, and the optimal dosage was determined using ROC curve analysis. To ascertain the minimal value for adequate fluorescence emission intensity, ROC curve analysis was performed to discriminate between binary outcomes: "strong" or "weak" emission. Furthermore, to establish the maximal value for maintaining a clear fluorescence border, ROC curve analysis was conducted to discriminate between "clear" and "contaminated" in the second evaluation.

## Results

A total of 21 patients were initially attempted for the positive staining method in laparoscopic ALR. Two cases were converted to the negative staining method due to difficulty in puncturing the tumor-bearing portal vein from the running blood vessel. In one case, ICG flowed into the hepatic vein, causing the entire liver to exhibit fluorescence; thus, the ICG fluorescence method was abandoned. Finally, eighteen patients were identified for analysis. Table 1 provides an overview of the baseline characteristics of the 18 patients. The most frequently used puncture method was percutaneous, employed in 15 cases. This was followed by LUS-guided and direct approaches, used in two and one cases, respectively.

**Table 1:** Baseline characteristics of the study population. CRLM, colorectal liver metastasis; HCC, hepatocellular carcinoma; CCC, cholangiocellular carcinoma; ML, malignant lymphoma; RAS, right anterior sectionectomy; RPS, right posterior sectionectomy; ICG, indocyanine green; LUS, laparoscopic ultrasonography; P5, portal branch of segment 5; P6, portal branch of segment 6; P7, portal branch of segment 7; P8, portal branch of segment 8; anterior, anterior branch of portal vein; posterior, posterior branch of portal vein.

Variables	All patients (n=18)
Sex, male/female	12/6
Age, years, median (range)	72 (51-85)
Indication HCC/CRLM/CCC, n	10/5/3
Operation segmentectomy/RAS/RPS, n	14/2/2
Approach percutaneous/LUS-guided/direct, n	15/2/1
Portal branch P5/P6/P7/P8/anterior/posterior, n	2/1/6/6/1/2
Fluorescence liver volume, mL, median (range)	160 (84-300)
ICG dosage, mg, median (range)	0.188 (0.025-0.5)
ICG dosage per volume, mg/100 mL, median (range)	0.063 (0.013-0.303)
Evaluation at 1st stage	
Strong/weak, n	14/4
Evaluation at 2nd stage	
Clear/contaminated, n	15/3

In terms of measurements of liver volume, the median fluorescence liver volume was 160 mL, with a range of 84-300 mL. The median ICG dosage was 0.188 mg, with a range of 0.025-0.5 mg. The median ICG dosage per liver volume was 0.063 mg/100 mL, with a range of 0.013 mg/100 mL to 0.303 mg/100 mL. 

Fluorescence intensity and border clarity evaluation

**Figure 1:**
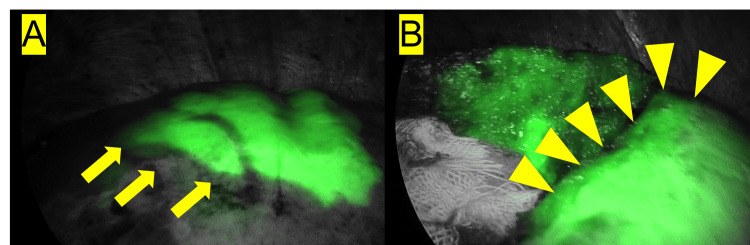
ICG fluorescence-guided identification of segment 8. A patient was administered an injection of indocyanine green (ICG) (0.303 mg/100 mL) targeting the portal branch of segment 8. Initial assessment (A) indicated a "strong" response, with the border appearing clearly defined (as indicated by the yellow arrow). However, during the surgery (B), the resection border became indistinct (denoted by the yellow arrowhead).

Of the 18 cases in which punctures were successfully performed, 14 were rated as "strong" and four as "weak" in the initial evaluation. During the subsequent stage of evaluation, 15 cases were deemed to have a "clear" result, while three were identified as "contaminated". For example, a patient who received an ICG (0.303 mg/100 mL) injection for segment 8 (S8). The initial evaluation was "strong"; however, the resection border turned out to be unclear during the operation, as shown in Figure 1.

**Figure 2:**
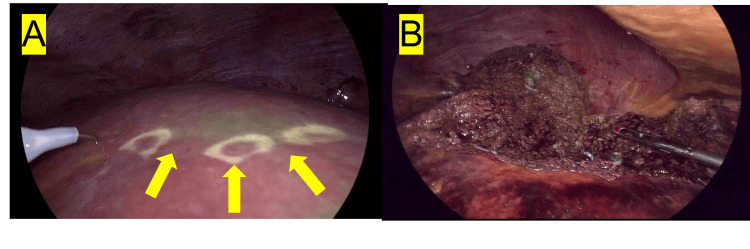
ICG fluorescence-guided segmentectomy of segment 8 with weak segmentation. A patient received an indocyanine green (ICG) injection (0.027 mg/100 mL) aimed at the portal branch of segment 8. The initial evaluation (A) showed a "weak" fluorescence response, but the boundary was clearly visible (as indicated by the yellow arrow). Despite this, during the surgery (B), the weak fluorescence segmentation remained constant without any contamination into other segments.

In contrast, a patient who underwent segmentectomy for S8 exhibited "weak" staining after being administered ICG (0.027 mg/100 mL). Nonetheless, no contamination occurred during the procedure, as shown in Figure 2.

**Figure 3:**
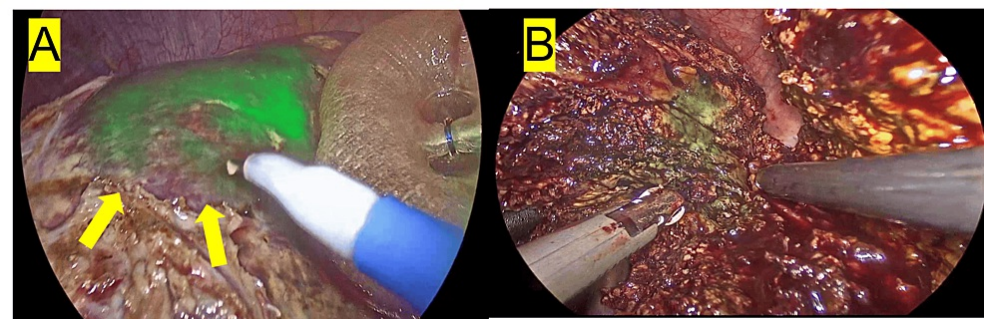
ICG fluorescence-guided segmentectomy of segment 7. A patient was administered an indocyanine green (ICG) injection (0.042 mg/100 mL) targeting the portal branch of segment 7. Upon initial assessment (A), a "strong" fluorescence response was observed, with the boundary appearing distinctly (as pointed out by the yellow arrow). Nevertheless, during the surgery (B), the border of fluorescence segmentation remained distinctly constant, with no contamination observed in the deeper parts of the liver parenchyma.

Lastly, for a patient, the fluorescence of segment 7 (S7) with ICG (0.042 mg/100 mL) was initially categorized as "strong". A clear border was consistently maintained throughout the resection, as shown in Figure 3.

Optimal ICG dosing determination

**Figure 4:**
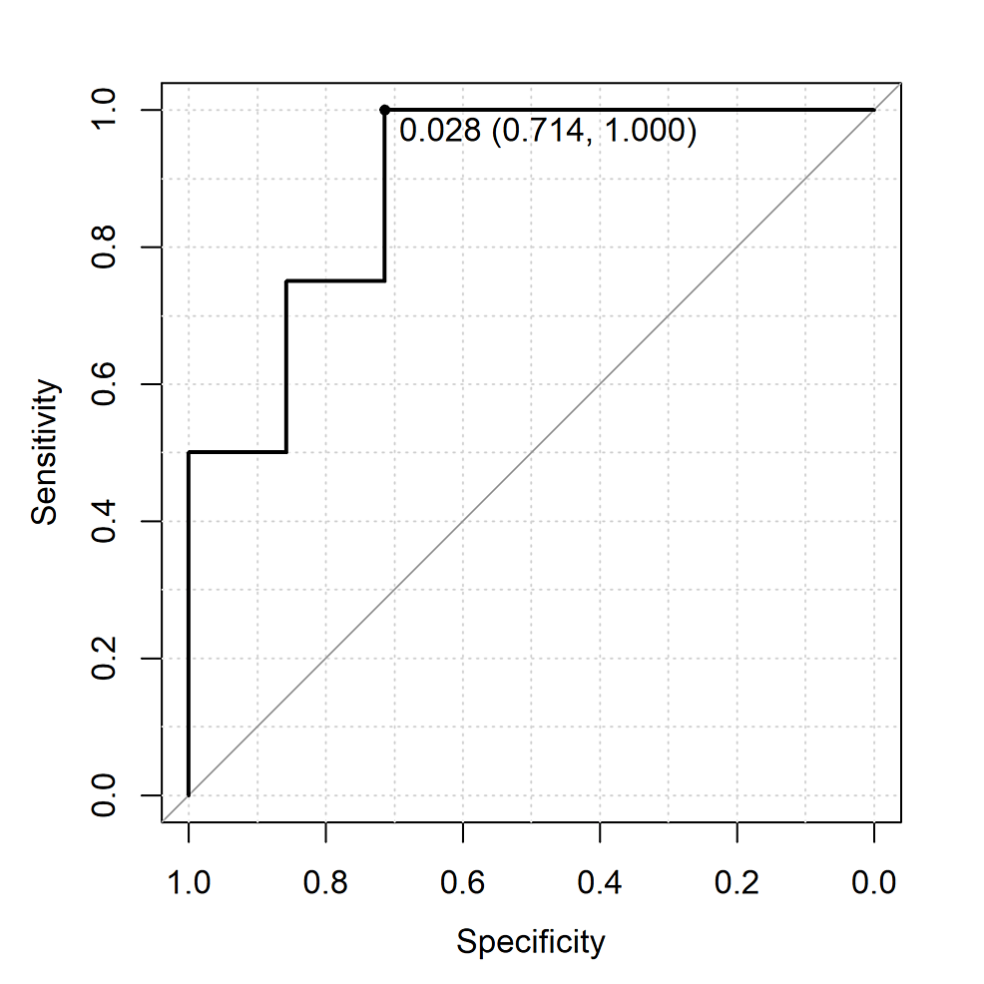
A receiver operating characteristic curve analysis to ensure sufficient fluorescence intensity. This figure presents the receiver operating characteristic curve analysis used to determine the minimum indocyanine green (ICG) dosage to achieve surgically sufficient fluorescence intensity. The analysis pinpointed an optimal ICG dosage of 0.028 mg/100 g, represented by an area under the curve (AUC) value of 0.893, a sensitivity of 100%, and a specificity of 71%, indicating a good balance between sensitivity and specificity.

An ROC curve analysis was conducted to identify the optimal dose of ICG that ensures adequate fluorescence intensity while maintaining clear borders during dissection. The analysis revealed that an ICG dose of 0.028 mg/100 g was optimal for achieving the minimum ICG dosage to achieve surgically sufficient fluorescence intensity (area under the curve [AUC]: 0.893, sensitivity: 100%, specificity: 71%) as shown in Figure 4.

**Figure 5:**
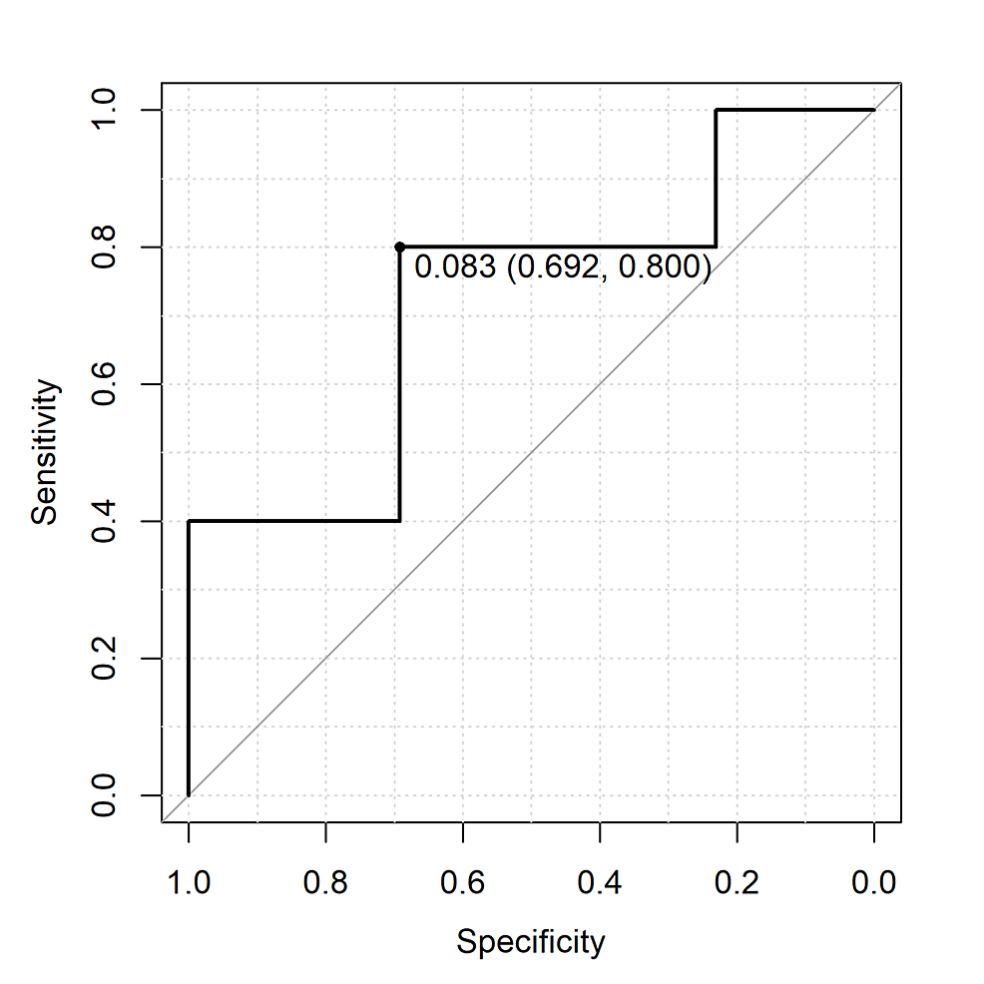
A receiver operating characteristic curve analysis to prevent the spread of fluorescence to non-target areas. The figure displays a receiver operating characteristic curve analysis, used to determine the optimal indocyanine green (ICG) dosage that prevents fluorescence from spreading to non-target areas until the completion of dissection. The analysis identifies an ICG dose of 0.083 mg/100 g as optimal, with an area under the curve (AUC) value of 0.723, a sensitivity of 80%, and a specificity of 69%, indicating a favorable balance between sensitivity and specificity.

On the other hand, a dose of 0.083 mg/100g or below prevented the spread of fluorescence to non-target areas until after dissection was complete (AUC: 0.723, Sensitivity: 80%, Specificity: 69%) as shown in Figure 5.

When ICG was administered at a dose between 0.028 and 0.083 mg/100 g, 11 out of 18 cases were correctly diagnosed with an accuracy of 62%.

## Discussion

ICG is a low-toxicity, near-infrared fluorescent dye that is quickly absorbed into the liver from the bloodstream and is primarily excreted into the bile. The excitation and emission wavelengths of ICG, which fall between 750 nm and 800 nm and between 805 nm and 845 nm respectively, enable the detection of vascular structures up to 1 cm below surfaces concealed within clots or liver parenchymal tissue. This is due to the superior translucency offered by the tissue's optical window (600-900 nm) [9]. It is known that ICG binds to lipoprotein in the blood to increase fluorescence intensity, although ICG alone does not exhibit strong fluorescence [10]. In an ex vivo environment, Hayashi et al. determined that the minimum fluorescence emission occurred at a 2.5 x 10^-4^ mg/mL ICG concentration in blood plasma, and the maximum emission occurred at 2.5 x 10^-2^ mg/mL [11]. The intensity of fluorescence coloration is directly proportional to ICG concentration but diminishes as the concentration exceeds a certain level (known as the quenching phenomenon) [11]. Upon the administration of ICG into the portal vein, hepatocytes selectively absorb it from the sinusoids before subsequently excreting it into the bile canaliculi [12]. This biological process is primarily determined by cell function, specifically the pigment uptake capacity of hepatocytes. However, the uptake of ICG is not uniformly executed by all hepatocytes due to innate variations in their functional capacities [13].

Presumably, residual ICG that remains unincorporated following the initial injection moves from the hepatic vein into the inferior vena cava, entering the systemic circulation during the positive staining method. From here, ICG is absorbed by hepatocytes scattered throughout the liver, including those in the non-tumor-bearing portal vein region. While this process is essential, it can sometimes result in fluorescence in undesired liver portal segments during liver resection. Theoretically, an ICG concentration below 2.5 x 10^-4^ mg/mL in blood plasma does not produce fluorescence [11]. For an individual weighing 60 kg (assuming they have 3 L of blood plasma), administering 0.25 mg of ICG would result in an ICG concentration of 8.3 x 10^-5^ mg/mL in the bloodstream. This concentration is below the threshold expected to induce fluorescence. However, based on our observations and supported by various published studies, injecting 0.25 mg of ICG often causes fluorescence throughout the liver, irrespective of whether the positive or negative staining method is employed [6]. This peculiar observation can be attributed to the fact that the ICG concentration absorbed by the hepatocytes might exceed the concentration calculated by simply dividing the amount in the blood plasma. Therefore, determining an optimal ICG dose for achieving a specific ICG concentration in the hepatocytes is clinically important and could significantly enhance the precision and effectiveness of ICG-assisted liver imaging.

The extensive variability in ICG dosages reported in the literature, as exemplified by Wakabayashi et al., ranges from 0.025 mg to 12.5 mg and can be attributed to the inclusion of open surgery, which is performed under more natural light, as opposed to the less naturally illuminated environment of laparoscopic surgery [6]. They disclosed that ten of the 21 studies involved open surgery with an ICG dose ranging from 0.25 mg to 12.5 mg per body, while the remaining eleven articles pertaining to minimally invasive surgery reported doses between 0.025 mg and 5 mg per body using the positive staining method. These findings suggest that laparoscopic liver resection may require lower ICG dosages compared to open liver resection. Consequently, it is imperative to determine the most appropriate ICG dosage, particularly for laparoscopic liver resection. Moreover, ICG doses have typically been reported on a per body basis; however, as the target liver volume increases, the required ICG dosage will inevitably rise. To address this issue, we calculated the liver volume of the target portal territory using CT volumetry, thus establishing an optimal concentration-to-liver volume ratio in accordance with real clinical practice.

As mentioned above, the quality of liver fluorescence enhancement is associated with liver functional capacity, which in a clinical setting includes biochemical panels such as the ICG clearance test at 15 minutes (ICG15R) and portal venous pressure. In our auxiliary analysis, ICG15R was not associated with the determination of optimal dosage. However, theoretically, these two factors should be associated with ICG clearance capacity. Further study involving not only ICG dosage but also these physiological factors is needed to assess adequate liver fluorescence visualization in laparoscopic surgery.

The present study has some limitations. Firstly, it is a retrospective study, and as such, unmeasured confounding factors may be inherent to the observational nature of the study. Moreover, the evaluation of fluorescence intensity was subjective, and quantitative determination remains a challenge due to equipment costs. Additionally, laparoscopic cameras from Storz and Stryker were utilized, and the excitation and fluorescence wavelengths may differ between manufacturers, potentially resulting in variations in fluorescence intensity despite identical ICG doses. Furthermore, our sample size of eighteen cases, while yielding an appropriate AUC of 0.7 for the ROC curve, may not be sufficiently large to draw definitive conclusions. Future investigations with larger cohorts are required to confirm the advantages of our findings.

## Conclusions

When employing the positive staining method for laparoscopic anatomical resections, it is essential to administer an optimal ICG dosage, which typically ranges between 0.028 and 0.083 mg for every 100 mL of liver volume. For each patient, this dosage should be meticulously determined, factoring in the expected liver volume for resection.

Adhering to this refined approach ensures that laparoscopic anatomical resections are conducted with greater accuracy and safety, further bolstering the overall security and efficacy of the surgical intervention for patients.
